# Time trends in access to smoking cessation support for people with depression or severe mental illness: a cohort study in English primary care

**DOI:** 10.1136/bmjopen-2020-048341

**Published:** 2021-12-03

**Authors:** Milena Falcaro, David Osborn, Joseph Hayes, Lisa Couperthwaite, Scott Weich, Kate R Walters

**Affiliations:** 1 Guy's Cancer Centre, King's College London, London, UK; 2 Faculty of Brain Sciences, University College London, London, UK; 3 McPin Foundation, London, UK; 4 School of Health and Related Research, The University of Sheffield, Sheffield, UK; 5 Department of Primary Care & Population Health, University College London, London, UK

**Keywords:** primary care, depression & mood disorders, schizophrenia & psychotic disorders, public health

## Abstract

**Objectives:**

To investigate delivery of smoking cessation interventions, recorded quit attempts and successful quitting rates within primary care in smokers with depression or severe mental illness (SMI) compared with those without.

**Design:**

Longitudinal cohort study using primary healthcare records.

**Setting:**

English primary care.

**Participants:**

882 849 patients registered with participating practices recorded as current smokers during 2007–2014, including three groups: (1) 13 078 with SMI, (2) 55 630 with no SMI but recent depression and (3) 814 141 with no SMI nor recent depression.

**Outcomes:**

Recorded advice to quit smoking, referrals to smoking cessation services, prescriptions for smoking cessation medication, recorded quit attempts and changes of smoking status.

**Results:**

The majority (>70%) of smokers had recorded smoking cessation advice. This was consistently higher in those with SMI than the other cohorts of patients, although the gap greatly reduced in more recent years. Increases in smoking cessation advice over time were not accompanied by increases in recorded attempts to quit or changes of smoking status. Overall nicotine replacement therapy prescribing by general practitioners (GPs) was higher in those with SMI (10.1%) and depression (8.7%) than those without (5.9%), but a downward time trend was observed in all groups. Bupropion and varenicline prescribing was very low and lower for those with SMI. Few smokers (<5%) had referrals to stop smoking services, though this increased over time, but no significant differences were observed between those with and without mental health problems.

**Conclusions:**

There was no evidence of consistent inequalities in access to GP-delivered smoking cessation interventions for people with mental health conditions. Smoking cessation advice was widely reported as taking place in all groups. In order to address the widening gap in smoking prevalence in those with poor mental health compared with those without, the emphasis should be on addressing the quality of advice and support given.

**Strengths and limitations of this study**

- This is, to our knowledge, the first nationally representative study evaluating trends over time in smoking cessation management practices in people with depression and severe mental illness compared with the general population in English primary care.
- Our data set includes robust information on smoking cessation prescriptions in primary care in England.
- The data set is however limited only to prescribed medications and does not include over-the-counter nicotine replacement therapy or e-cigarette use.
- There is likely to be under-recording of smoking quit attempts and smoking status changes in primary care records.

## Introduction

Poor mental health has been shown to be the most common single cause of disability-adjusted life years lost globally.^1^ Studies have found that people with mental ill-health have elevated mortality rates^2 3^ and that for those with severe mental illness (SMI; schizophrenia, bipolar disorder and other non-organic psychoses) this mortality gap has been widening over the last decade in the UK.^4^ Smoking is often seen as a means of coping with stress and is more common in those with poor mental health.^5–7^ Prevalence rates of smoking were 34.1% in those with common mental disorders compared with 19.6% in those without mental health conditions in 2014,^8^ and higher rates are seen in those with SMI (58% in men and 41% in women in the UK).^7^ However, it is a key modifiable risk factor which greatly contributes to the excess risk of cardiovascular diseases (CVD) and mortality observed in this population. It is therefore an important target for intervention. There is consistent evidence that quitting smoking leads to improvements in both mental and physical health.^9–11^ It has also been shown that those with mental illness can successfully quit smoking with tailored support.^8^ Effectiveness can be similar to the general population,^12 13^ with stronger effects seen in those with current depression in studies with a pharmacological smoking cessation treatment than those with psychological treatments.^13^ However, most of these trials have been conducted in specialist clinic settings in stable populations and findings for longer term outcomes are more mixed,^13 14^ for example, in the SCIMITAR+ trial improved quit rates at 6 months are not sustained by 12 months in people with SMI.^14^

Since people with mental illnesses are in regular contact with their general practice and this is where more assertive management of CVD risk factors—including smoking cessation—may occur, general practitioners (GPs) can play a key role in incentivising and helping patients to stop smoking. There is however to our knowledge little literature that has tested the effectiveness of interventions for smoking cessation in primary care settings for those with mental health problems, with no trials in those with SMI and only one in depression.^12 13^ It is therefore important to assess the management of smoking cessation for people with poor mental health in this setting, including both smoking cessation advice and evidence-based treatments, and to both carefully document patterns of receipt over time and identify areas for improvements. Previous research exploring this has been limited. A cross-sectional study using English primary care data in a single year (July 2009–June 2010) reported that around one-half of smokers with a mental health diagnosis (including SMI and depression, among others) received smoking cessation advice from their GP in that year compared with around one-third of smokers without a mental health diagnosis.^15^ This suggests that GPs may promote smoking cessation more often in those with mental illness, though the content of this advice is unknown. Smoking prevalence is changing over time, reducing from 29.3% to 19.6% in those without a mental health condition and from 44.6% to 34.1% in those with a common mental disorder (depression and anxiety disorders) between 1993 and 2014 in Great Britain,^8^ the latter remaining therefore much higher than in the general population.^8 16 17^ Furthermore, the gap in CVD-related mortality for those with mental illness has also been rising over time,^4^ increasing the need to better understand primary care management of smoking cessation in people with SMI and to inform strategies to promote smoking cessation in people with poor mental health.

In this study we examined whether, at the point of recording or updating smoking status to reflect current smoking (ie, when there was an opportunity for smoking interventions to be implemented), GPs were more or less likely to offer smoking cessation interventions to those with depression or SMI compared with those without these conditions. We also investigated if this has changed over time and explored recorded quit attempts and successful quitting.

## Methods

### Study design

Longitudinal cohort study using healthcare records from English primary care.

### Data source

We used electronic health records from the English GP practices that contributed data to The Health Improvement Network (THIN), a large primary care database in the UK. Only data that met quality assurance standards^18^ were retained for analysis. THIN holds information on for example symptoms, diagnoses, referrals, prescribing, health indicators (eg, smoking status) and sociodemographic status. In particular, smoking status has been increasingly better recorded^19^ and is now routinely collected by many GPs as part of the Quality Outcomes Framework (QOF,^20^ a pay-for-performance scheme which financially rewards general practices for achieving specific targets across a range of indicators. Marston *et al*^21^ reported that around 84% of patients have had their smoking status recorded at least once during the first year of registration with a GP practice. THIN contains records on the delivery of smoking cessation advice and on quit attempts, although recorded quit attempts are likely to be incomplete as many will attempt this without seeing their GP. Data on smoking cessation pharmacotherapy are available for drugs prescribed by GPs. The first-line medicines for quitting smoking are nicotine replacement therapy (NRT), bupropion and varenicline. The latter two are solely issued on prescription, so their recording is complete. In contrast, NRT can be bought over the counter and, since information about these purchases is not captured in THIN, our findings will be confined to NRT prescribing within primary care. The lack of data on over-the-counter medication in THIN also means that the use of e-cigarettes is also not recorded as these electronic devices are not available on prescription in the UK.

### Study population

We included all patients aged 18 years or over who were permanently registered for at least 6 months with a participating practice between 1 January 2007 and 30 June 2015 and had their smoking status updated to ‘current smoker’ at least once between the start of the follow-up and 6 months from the end of it. This was chosen as it indicated both a contemporaneous record of their smoking status and an opportunity for the GP to deliver a smoking cessation intervention to that patient. If a patient had multiple updates during the study period, one of them was selected at random and treated as the index event. We restricted the sample to those with at least 6 months of follow-up time to allow time for the outcomes to be recorded. The beginning of the follow-up was set at the latest of the following: 1 January 2007, the patient’s 18th birthday, the patient’s date of registration with the practice and 1 year after the date the practice achieved an acceptable level of data quality.^18^ The end of the follow-up was the earliest of 30 June 2015, the patient’s transfer out of the practice, the patient’s date of death or the last date the practice contributed data to THIN.

### Outcomes

We considered outcomes to investigate the delivery of smoking cessation interventions and any evidence for the potential effectiveness of these interventions in terms of quit attempts and changes of smoking status. Specifically, we looked at whether or not patients had entries in their electronic health records for

1. advice to quit smoking,
2. referrals to stop-smoking services,
3. prescriptions for smoking cessation medication (NRT, varenicline or bupropion),
4. recorded quit attempts,
5. changes of smoking status to ex-smoker or non-smoker within 6 months from the index event, that is, the date (or the randomly selected one in case of multiple dates) when the smoking status was recorded as current smoker for that individual. Prescriptions for smoking cessation medications were identified using the relevant British National Formulary codes.^22^ The read code lists for the other four types of outcome were created using standard methods^23^ and were approved by a GP. Specifically, quit attempts were identified using codes for changes of smoking status to ‘ex-smoker’ or ‘non-smoker’, clear quit attempts (eg, ‘Recently stopped smoking’), referrals to stop-smoking services (excluding codes related to referrals which were merely offered or declined) and codes indicating that the patient was being prescribed drug treatment for smoking cessation.

### Recorded mental health status

Smokers were grouped into three cohorts of patients depending on whether they had

1. a history of SMI, that is, a record of SMI diagnosis at any time in their healthcare record,
2. no history of SMI but recent recorded diagnoses or symptoms of depression,
3. no history of SMI nor recent recorded diagnoses or symptoms of depression.

People with a history of SMI were identified using read codes for diagnoses of bipolar disorder, schizophrenia and other non-organic psychotic illnesses (eg, delusional disorder) or indicating the inclusion on the SMI register. Individuals were considered as having had recent episodes of depression if during the year prior to the index date, or since their registration with the practice if later, they had at least one entry in their medical records for symptoms or diagnoses of depression. Read codes related to history of depression were excluded to avoid historical episodes of depression being treated as incident cases.

### Statistical analysis

We calculated the proportion of people who had the outcomes of interest during the 6-month window following the index date. These proportions, along with 95% CIs, were stratified by mental health group and were derived in relation to the calendar year in which the index smoking status update was recorded. Since data from patients attending the same GP practice are likely to be correlated, the 95% CIs were calculated using robust standard errors^24 25^ to account for the data clustering.

We also used direct standardisation^26^ to remove differences in the age and sex distributions across the three groups of patients. Specifically, age was categorised into eight groups (18–24, 25–34, 35–44,…, 75–84 and 85+) and the cohort of patients with no history of SMI nor depression was treated as the reference population. The standardisation was carried out separately for each calendar year.

All analyses were performed using Stata V.14.^27^

### Patient and public involvement

People with lived experience (including LC, co-author) and a support worker from the McPin Foundation were involved from the inception of the study, including informing the idea, the design and protocol for the funder. They actively contributed throughout, attending study meetings, contributing to the interpretation of the findings and commenting on drafts of the paper. This included highlighting ongoing misconceptions around the safety of varenicline/bupropion in people with mental health problems and the importance of the quality of advice given for smoking cessation in the context of ongoing mental health symptoms.

## Results

Between 1 January 2007 and 31 December 2014, there were 882 849 patients who met the inclusion criteria and had at least one smoking status update to current smoker. Table 1 reports descriptive statistics for these patients, stratified by mental health condition. Smokers with a history of SMI tended to be older than other smokers.

**Table 1:** Descriptive statistics for the smokers who entered our study

	History of SMI	Depression	No SMI nor depression
Number of patients (row %)	13 078 (1.5%)	55 630 (6.3%)	814 141 (92.2%)
Gender			
Male	7325 (56.0%)	21 439 (38.5%)	431 743 (53.0%)
Female	5753 (44.0%)	34 191 (61.5%)	382 398 (47.0%)
Age at index date			
Median	45.1	37.7	39.5
IQR	(35.4–56.1)	(27.2–48.5)	(27.8–53.1)
Interventions within 6 months from index date			
% Advised to quit	80.0%	73.1%	70.3%
% With referrals to stop smoking services	2.5%	2.5%	2.2%
% With prescribed antismoking drugs medication:			
NRT	10.1%	8.7%	5.9%
Bupropion	0.2%	0.4%	0.4%
Varenicline	1.3%	2.9%	3.4%
Evidence of attempts to quit within 6 months from index date			
% With recorded quit attempts	18.3%	18.2%	16.3%
% With smoking status change	5.7%	5.9%	6.4%

In case a patient had more than one smoking status update to ‘current smoker’ during the follow-up, we selected one of them at random and used it as the index event.

NRT, nicotine replacement therapy; SMI, severe mental illness.

### Smoking cessation interventions

**Figure 1:**
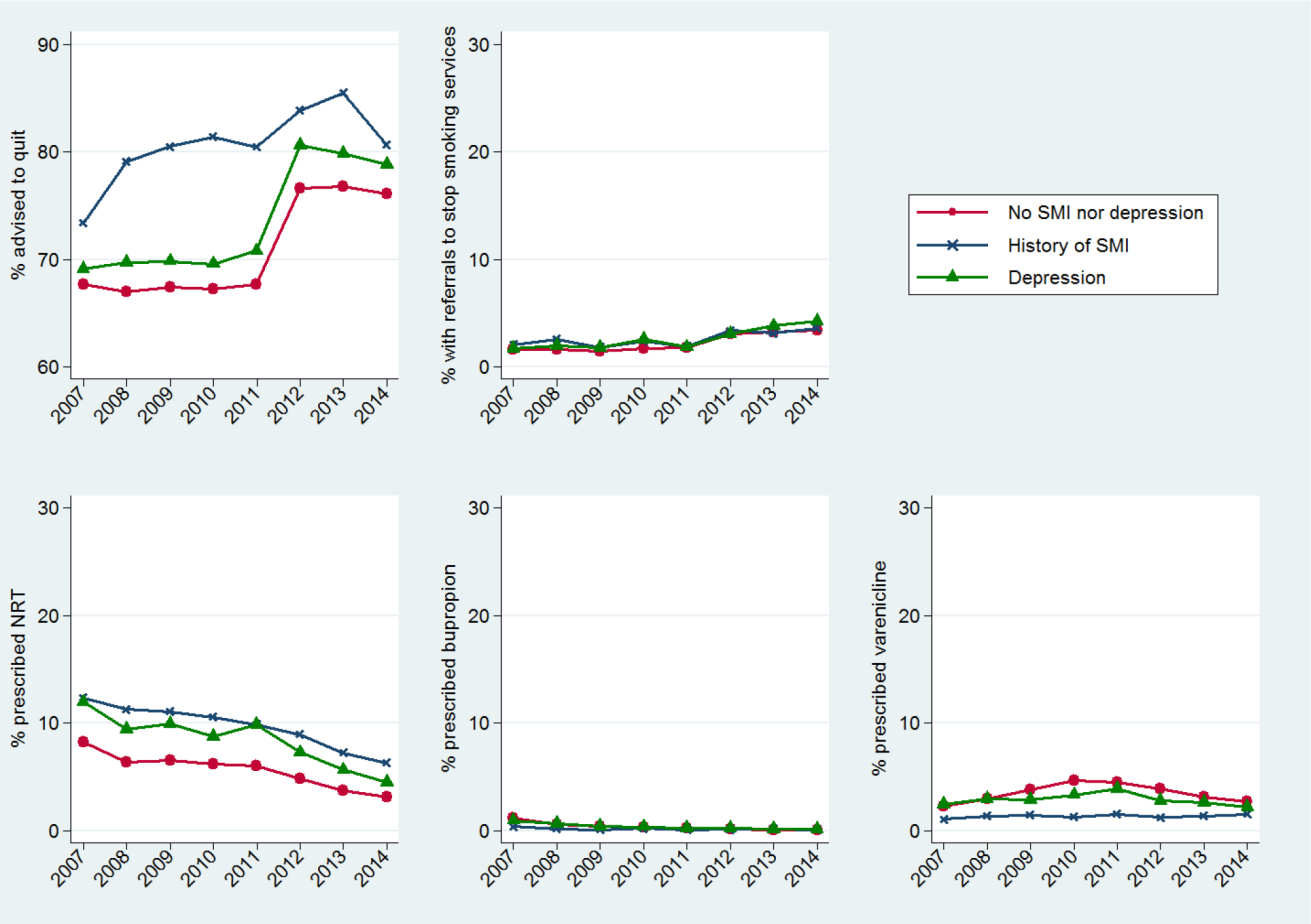
Annual proportions of smokers who received smoking cessation interventions within 6 months from the index smoking status update. Proportions are plotted by mental health condition and in relation to the calendar year in which the index smoking status update was recorded. SMI, severe mental illness.

The proportions of patients who were advised to quit, received referrals to stop-smoking services or were prescribed cessation medication within 6 months from the index date are displayed in figure 1 by calendar year and mental health group. The corresponding numeric values and 95% CIs are reported in table 2. Age and gender standardisation of these proportions did not lead to significantly different findings so, for simplicity, only the unstandardised results are reported hereafter.

**Table 2:** Annual proportions (and 95% CIs) of smokers who received smoking cessation interventions within 6 months from the index date

	Advice to quit smoking	Referrals to smoking cessation services	Smoking cessation drug therapy		
	% (95% CI)	% (95% CI)	NRT % (95% CI)	Bupropion % (95% CI)	Varenicline % (95% CI)
**History of SMI**					
**2007**	73.39 (70.88 to 75.77)	2.09 (1.50 to 2.88)	12.34 (11.05 to 13.76)	0.38 (0.20 to 0.71)	1.04 (0.65 to 1.66)
**2008**	79.07 (76.79 to 81.18)	2.57 (1.50 to 4.37)	11.26 (9.78 to 12.92)	0.16 (0.05 to 0.51)	1.37 (0.91 to 2.04)
**2009**	80.48 (78.22 to 82.56)	1.79 (1.10 to 2.90)	11.07 (9.74 to 12.56)	0.06 (0.01 to 0.40)	1.45 (0.97 to 2.17)
**2010**	81.37 (78.89 to 83.62)	2.39 (1.64 to 3.48)	10.54 (9.06 to 12.24)	0.19 (0.06 to 0.60)	1.29 (0.76 to 2.18)
**2011**	80.43 (77.81 to 82.81)	1.88 (1.24 to 2.82)	9.85 (8.31 to 11.64)	0.07 (0.01 to 0.48)	1.54 (1.05 to 2.27)
**2012**	83.83 (81.57 to 85.85)	3.37 (2.31 to 4.89)	8.95 (7.42 to 10.75)	0.14 (0.03 to 0.54)	1.24 (0.79 to 1.95)
**2013**	85.47 (83.27 to 87.42)	3.12 (2.13 to 4.54)	7.23 (5.98 to 8.71)	0.14 (0.04 to 0.56)	1.35 (0.85 to 2.12)
**2014**	80.60 (77.61 to 83.28)	3.62 (2.41 to 5.40)	6.29 (4.95 to 7.96)	0.09 (0.01 to 0.61)	1.55 (1.00 to 2.40)
**Depression**					
**2007**	69.14 (67.39 to 70.83)	1.69 (1.29 to 2.21)	11.94 (11.08 to 12.86)	0.95 (0.78 to 1.16)	2.44 (2.03 to 2.94)
**2008**	69.71 (67.87 to 71.49)	1.98 (1.42 to 2.77)	9.40 (8.55 to 10.33)	0.68 (0.51 to 0.90)	2.94 (2.49 to 3.48)
**2009**	69.83 (68.02 to 71.58)	1.81 (1.43 to 2.30)	9.96 (9.12 to 10.86)	0.38 (0.26 to 0.55)	2.87 (2.44 to 3.36)
**2010**	69.58 (67.68 to 71.42)	2.58 (1.97 to 3.37)	8.74 (7.84 to 9.73)	0.36 (0.23 to 0.56)	3.34 (2.91 to 3.82)
**2011**	70.78 (68.93 to 72.57)	1.85 (1.32 to 2.60)	9.83 (8.87 to 10.87)	0.25 (0.15 to 0.41)	3.88 (3.35 to 4.48)
**2012**	80.60 (79.09 to 82.02)	3.09 (2.34 to 4.06)	7.27 (6.55 to 8.07)	0.24 (0.15 to 0.39)	2.80 (2.42 to 3.24)
**2013**	79.83 (78.26 to 81.32)	3.80 (2.95 to 4.87)	5.65 (4.96 to 6.42)	0.16 (0.09 to 0.28)	2.65 (2.23 to 3.15)
**2014**	78.81 (76.79 to 80.70)	4.28 (3.19 to 5.72)	4.51 (3.79 to 5.36)	0.13 (0.06 to 0.28)	2.21 (1.80 to 2.72)
**No SMI nor depression**					
**2007**	67.63 (66.10 to 69.12)	1.60 (1.31 to 1.96)	8.26 (7.81 to 8.73)	1.16 (1.06 to 1.26)	2.26 (1.99 to 2.57)
**2008**	66.97 (65.45 to 68.46)	1.65 (1.26 to 2.16)	6.38 (5.99 to 6.78)	0.60 (0.52 to 0.69)	2.98 (2.71 to 3.28)
**2009**	67.43 (65.93 to 68.90)	1.47 (1.23 to 1.76)	6.50 (6.11 to 6.92)	0.43 (0.36 to 0.50)	3.77 (3.49 to 4.07)
**2010**	67.22 (65.81 to 68.59)	1.73 (1.34 to 2.22)	6.20 (5.79 to 6.63)	0.33 (0.27 to 0.41)	4.62 (4.32 to 4.94)
**2011**	67.65 (66.05 to 69.21)	1.80 (1.40 to 2.31)	6.06 (5.64 to 6.50)	0.24 (0.19 to 0.30)	4.51 (4.22 to 4.82)
**2012**	76.56 (75.21 to 77.87)	3.09 (2.41 to 3.94)	4.86 (4.49 to 5.25)	0.14 (0.10 to 0.18)	3.87 (3.62 to 4.14)
**2013**	76.78 (75.32 to 78.19)	3.21 (2.50 to 4.12)	3.70 (3.41 to 4.02)	0.11 (0.08 to 0.14)	3.12 (2.89 to 3.36)
**2014**	76.11 (74.5 to 77.66)	3.43 (2.62 to 4.48)	3.12 (2.82 to 3.45)	0.09 (0.07 to 0.12)	2.71 (2.48 to 2.96)

Proportions are reported by mental health condition and in relation to the calendar year in which the index event was recorded.

NRT, nicotine replacement therapy; SMI, severe mental illness.

#### Advice to quit smoking

The majority (>70%) of the smokers who entered our study received advice to quit within 6 months from the index smoking status update, with the proportion reaching 80% among those with a history of SMI. When we looked at trends over time we observed similar patterns in the annual percentages of smokers advised to quit among those with depression and those with no mental health problems, with a steep increase between 2011 and 2012. The corresponding percentages for people with SMI were consistently higher than in the other two cohorts of patients across the whole study period but differences greatly reduced after 2012.

#### Referrals to smoking cessation services

The proportion of current smokers recorded as being referred to stop-smoking services was very low (<5%). A small increase was observed over time, with the proportion for example for those with depression increasing from 1.7% in 2007 to 4.3% in 2014, but no significant differences were found when comparing those with SMI or depression and those without.

#### Smoking cessation pharmacotherapy

Overall, NRT was the medication most commonly prescribed for smoking cessation within primary care. The proportion of current smokers who had prescriptions for NRT during the 6 months from when their smoking status was updated was 10.1% among the patients with a history of SMI, 8.7% among those with depression and 5.9% among the general population without depression or SMI. In contrast, rates of varenicline or bupropion prescribing were very low, especially in the SMI group. When we examined temporal changes of pharmacotherapy prescribing, we observed a clear decline over time in the proportion of smokers who were prescribed NRT by their GP, with rates of prescribing of NRT consistently higher in those with SMI and depression than the general population without these conditions.

### Evidence of attempts to quit smoking

**Figure 2:**
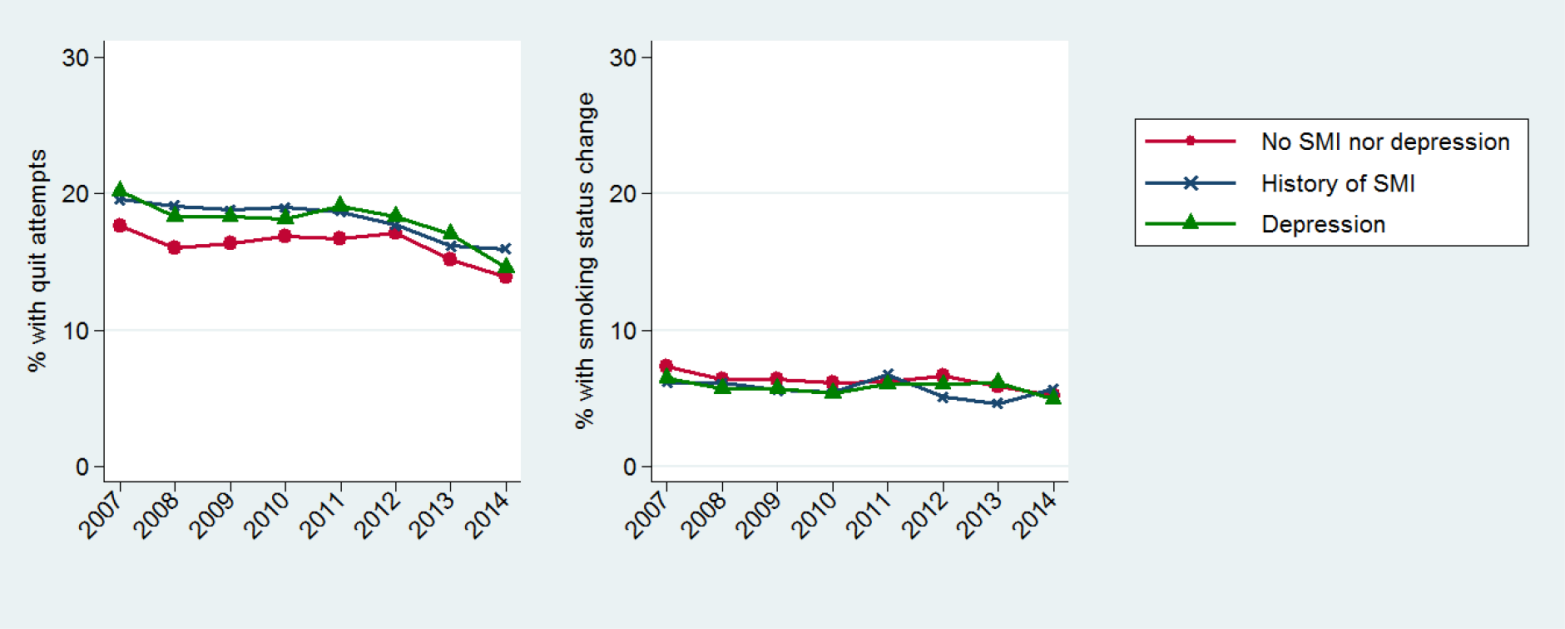
Annual proportions of smokers with quit attempts or changes of smoking status recorded within 6 months from the index smoking status update. Proportions are plotted by mental health condition and in relation to the calendar year in which the smoking status update was recorded. SMI, severe mental illness.

The proportions of smokers for whom we have evidence of quit attempts or who had a change of smoking status during the 6 months following their smoking status update are displayed in figure 2 and are reported in table 3 along with the 95% CIs. Age-standardised and sex-standardised proportions were derived but they did not significantly alter our findings so, as for the smoking cessation interventions, they are here omitted.

**Table 3:** Annual proportions (and 95% CIs) of smokers with quit attempts or a change of smoking status recorded within 6 months from the index smoking status update

	Recorded quit attempts	Change of smoking status
**History of SMI**		
**2007**	19.56 (17.98 to 21.24)	6.13 (5.13 to 7.31)
**2008**	19.07 (17.05 to 21.27)	6.12 (4.98 to 7.50)
**2009**	18.79 (16.82 to 20.94)	5.59 (4.68 to 6.67)
**2010**	18.95 (16.90 to 21.19)	5.43 (4.36 to 6.75)
**2011**	18.63 (16.62 to 20.83)	6.77 (5.63 to 8.12)
**2012**	17.69 (15.55 to 20.05)	5.09 (4.09 to 6.33)
**2013**	16.16 (14.05 to 18.52)	4.61 (3.61 to 5.86)
**2014**	15.95 (13.69 to 18.50)	5.69 (4.47 to 7.22)
**Depression**		
**2007**	20.20 (19.13 to 21.31)	6.46 (5.87 to 7.11)
**2008**	18.32 (17.09 to 19.61)	5.73 (5.21 to 6.30)
**2009**	18.29 (17.17 to 19.47)	5.70 (5.10 to 6.38)
**2010**	18.15 (16.95 to 19.42)	5.38 (4.84 to 5.96)
**2011**	19.09 (17.90 to 20.34)	6.01 (5.43 to 6.65)
**2012**	18.29 (17.09 to 19.55)	6.02 (5.46 to 6.63)
**2013**	17.02 (15.73 to 18.40)	6.15 (5.61 to 6.75)
**2014**	14.59 (13.18 to 16.12)	4.92 (4.31 to 5.61)
**No SMI nor depression**		
**2007**	17.64 (16.97 to 18.34)	7.31 (7.01 to 7.63)
**2008**	15.97 (15.20 to 16.77)	6.36 (6.06 to 6.66)
**2009**	16.36 (15.66 to 17.08)	6.36 (6.07 to 6.65)
**2010**	16.82 (16.11 to 17.56)	6.15 (5.85 to 6.46)
**2011**	16.68 (16.00 to 17.37)	6.22 (5.91 to 6.55)
**2012**	17.14 (16.26 to 18.06)	6.62 (6.34 to 6.92)
**2013**	15.19 (14.32 to 16.10)	5.91 (5.64 to 6.20)
**2014**	13.84 (12.84 to 14.90)	5.22 (4.95 to 5.50)

Proportions are reported by mental health condition and are derived in relation to the calendar year in which the index date falls.

SMI, severe mental illness.

#### Recorded quit attempts

Overall, smokers with recognised poor mental health were more likely than those without mental ill-health to have at least one quit attempt (defined by either codes of quit attempts or other evidence of attempted quitting, such as the prescription of smoking cessation medication) recorded during the 6 months after the index date. This was also observed across all the time points, although the gap reduced towards the end of the study period. In all three cohorts of smokers, the annual proportions of people with recorded quit attempts showed a reduction after 2012. In a supplementary analysis where we investigated the single components defining a recorded quit attempt we found that this reduction was mainly driven by the decline in NRT prescribing by GPs over this time.

#### Changes of smoking status

Changes of smoking status recorded within 6 months from the index smoking status update were observed in 5.7% of smokers with SMI as compared with 5.9% among those with depression and 6.4% among the other smokers. The time trends of these proportions remained fairly stable and did not show large differences across the three cohorts of smokers.

## Discussion

Our study shows that most GPs recorded providing smoking cessation advice to patients who are identified as current smokers, although the duration and quality of the advice provided cannot be determined from our data. Rates of recorded smoking cessation advice in UK primary care were high for all three groups of patients and highest in those with SMI. However, increases in smoking cessation advice over time were not accompanied by increases in the prescription of smoking cessation medication, recorded quit attempts or changes in smoking status. Few people (<5%) were recorded as being referred to smoking cessation services, with a small upward trend over time, but no significant differences were observed between those with and without mental health conditions. NRT prescribing by GPs was higher in smokers with SMI and depression, although it declined over time in all groups. Varenicline and bupropion prescribing was very low, and less common in those with SMI.

There were temporal trends in recorded smoking cessation interventions in primary care. We observed a steep increase in recorded advice between 2011 and 2012, particularly for those without SMI (including those with depression). The percentage of smokers with SMI recorded as having received smoking cessation advice was consistently higher than in the other two cohorts of patients across the whole study period but differences observed greatly reduced after 2012. Studies in the USA have similarly found higher rates of smoking cessation advice given to smokers with SMI.^28^ The increase in advice to quit smoking given to non-SMI populations post 2012 in our study may be due to changes in incentivisation of recording of smoking advice in England. Before 2012, the QOF incentives scheme rewarded GPs for providing smoking cessation advice but this was only in relation to smokers with specific medical conditions, which included those with SMI (but not depression) from 2006. In April 2012, a new incentive was introduced to encourage GPs to provide smoking cessation support and treatment to all current smokers aged 15 years or over, regardless of their medical history. This incentivisation seems to have worked to increase the recording (and one presumes delivery) of smoking cessation advice by GPs and recorded referrals to smoking cessation services.^29^ However, our findings demonstrated no corresponding rise in prescribing of smoking cessation medication, quit attempts or changes in smoking status over the same time period, suggesting that this rising reported cessation advice was not effective in influencing quit rates. From our data we cannot determine the quality of advice given, but it is likely to be brief when opportunistic, and qualitative research has found that it can be confrontational in approach.^30^ Previous research has also shown that primary care physicians are more likely to ‘Ask’ (ask all patients about tobacco use) and ‘Advise’ (advise all tobacco users to quit) than they are to ‘Assess’ (assess the willingness to quit), ‘Assist’ (assist with quitting) or ‘Arrange’ (arrange follow-up) as part of the 5A’s strategy to support smoking cessation.^31^ Our findings support that these latter steps—assessing motivation to quit, providing assistance and follow-up—may be key missing steps in supporting people with mental health conditions to quit.^32^

The number of recorded quit attempts in our study had, if anything, a downward trend. However, this appeared to be explained by reducing rates of NRT prescribing by GPs, which in turn may be driven by a corresponding rise in NRT uptake outside of primary care and increasing popularity of e-cigarettes. Our data source does not contain information on over-the-counter medication. Previous research has shown that the use of e-cigarettes in smokers increased between 2011 and 2013 and then levelled off afterwards.^33^ Beard *et al* reported that, after adjusting for confounding, the use of e-cigarettes for quitting was negatively associated with NRT on prescription.^34^ We found that NRT prescribing was higher in those with depression and SMI than the general population without these conditions, which could be explained by corresponding lower prescribing of bupropion or varenicline (in those with SMI) or by higher attendance rates at GPs in these groups (increasing accessibility to NRT via GPs) or higher levels of unemployment and associated free prescriptions for NRT which they may otherwise need to purchase.

In all three groups in our study, the proportion of people who changed their recorded smoking status from current smoker to ex-smoker was lower in 2014 than in 2007 suggesting that, while other evidence indicates uptake of smoking is going down (proportions of never smokers are increasing over time in England)^35^ thus reducing overall smoking prevalence, in those that do smoke slightly fewer are quitting than in previous years. We found no major differences in changes of recorded smoking status from current to ex-smoker (indicating a successful quit attempt) in those with mental health conditions compared with the general population, which is in contrast to other evidence suggesting quit rates may be lower in those with SMI.^36^ This might be explained by an under-recording of smoking status changes in those without mental health conditions as they visit their GP less frequently.

Rates of prescribing of varenicline and bupropion were very low overall, and even lower for those with SMI, despite good evidence of effectiveness of both drugs in those with SMI in increasing medium-long term quit rates^37 38^ and some evidence that varenicline may be more effective than NRT.^39 40^ Low prescribing probably reflects concerns among GPs and patients regarding the side effects of these two drugs during this period. In 2007, the US Food and Drug Administration (FDA) announced investigations into the safety of varenicline following concerns related to suicidality and other neuropsychiatric adverse events. Similar concerns were subsequently raised for bupropion too, prompting the FDA to issue in 2009 a black-box warning (the FDA’s most serious type of warning) for both varenicline and bupropion. Subsequent studies including large scale trials found no evidence to support these concerns^36 41 42^; however, the FDA warning for possible serious neuropsychiatric adverse events in patients quitting smoking was not removed until December 2016. Information campaigns may be needed for both smokers with mental health conditions and primary care providers to address this and encourage a greater uptake of prescribing of these drugs in people with mental health problems.

To our knowledge, this is the first study to explore time trends across a range of primary care management strategies for smoking cessation in people with mental health conditions using a large nationally representative dataset. As a study of routinely collected primary care data there are some inherent limitations, the key one being that we were only able to include pharmacotherapy prescribed by GPs and had no information on over-the-counter treatments, including the rising use of e-cigarettes. There is evidence however that recorded prescriptions for smoking cessation medication are comparable to national dispensing data and are a valid source for monitoring trends in prescribing for smoking cessation.^43^ We are only able to report on recorded data, and some interventions (eg, advice) may occur but are not entered into the patient’s record. However, information on prescription is of high quality in THIN and recorded advice levels were very high, suggesting that this was not a significant problem, though changes in recording practices over time may explain some of the patterns we observed. The prescribing records in our data source represent prescriptions issued by GP practices, and patients may not go on to take these medications. We included the issue of smoking cessation medication as an indicator of a ‘quit attempt’ (an intention to try to quit) and this may overestimate true quit attempts as not all patients will actually carry on to try stopping smoking. It is quite likely, nonetheless, that quit attempts were under-recorded as many people will not report this to their GP. Changes to smoking status will also depend on the GP becoming aware of this during the follow-up period, meaning that these may also be under-recorded. As those with mental health conditions visit their GP more often, there are more opportunities for them to receive smoking cessation support in this setting and it is possible that this explains the slightly higher recorded NRT prescribing observed in this group. This is not necessarily an indicator of better quality smoking cessation support in these populations.

Our results show that while smoking cessation advice is both very high and recorded as being offered more frequently in those with SMI this gap has narrowed in more recent years (probably due to changes in how recording of smoking cessation advice is incentivised), and changes in levels of advice is not linked to corresponding changes in quit attempts or smoking status. This is of concern given the ongoing high smoking rates in those with poor mental health, particularly those with SMI.^44^ There is some evidence that better quit rates can be achieved when smoking cessation is integrated within specialist mental healthcare settings^45^ or with the addition of spirometry and feedback on lung age and degree of airways obstruction.^46^ Our findings suggest that in order to close the widening gap in smoking prevalence and its consequences for people with mental health conditions, the focus in primary care should change from the quantity of advice given to the quality. This should include routine assessment of willingness to change, greater provision of assistance to quit and arranging follow-up as part of a ‘5As’ strategy^31^ and could include other elements such as spirometry with feedback to better support smoking cessation in people with mental health conditions. Incentivisation schemes for GPs should reflect this, in particular as due to funding restraints this is often the main source of smoking cessation support for people with mental health conditions.

## Conclusions

Recorded smoking cessation advice delivered by GPs was very high at times when GPs reviewed an individual’s smoking status, and higher in those with SMI. We found no evidence of consistent inequalities in access to smoking cessation interventions in primary care for people with recognised mental illness. In order to address the widening gap in smoking prevalence in those with poor mental health compared with those without, the emphasis should now be on addressing the quality of advice and support provided in primary care.

## Supplementary Material

Reviewer comments

Author's manuscript

## Data Availability

Data may be obtained from a third party and are not publicly available. Read code lists are available from the corresponding author at k.walters@ucl.ac.uk. No additional data available due to the nature of the dataset (healthcare records).
