# Correction: Time trends in access to smoking cessation support for people with depression or severe mental illness: a cohort study in English primary care

**DOI:** 10.1136/bmjopen-2020-048341corr1

**Published:** 2023-02-14

Falcaro M, Osborn D, Hayes J, *et al*. Time trends in access to smoking cessation support for people with depression or severe mental illness: a cohort study in English primary care. *BMJ Open* 2021;11:e048341. doi: 10.1136/bmjopen-2020-048341

The authors want to alert the readers about the below changes:

One author was removed from the paper at their request, and the patient and public involvement box and the contributors section have been edited to reflect this. The version displayed will be the latest version.

